# Factors Associated with Esophageal Candidiasis and Its Endoscopic Severity in the Era of Antiretroviral Therapy

**DOI:** 10.1371/journal.pone.0058217

**Published:** 2013-03-26

**Authors:** So Nishimura, Naoyoshi Nagata, Takuro Shimbo, Naoki Asayama, Junichi Akiyama, Norio Ohmagari, Hirohisa Yazaki, Shinichi Oka, Naomi Uemura

**Affiliations:** 1 Department of Gastroenterology and Hepatology, National Center for Global Health and Medicine, Kohnodai Hospital, Chiba, Japan; 2 Department of Gastroenterology and Hepatology, National Center for Global Health and Medicine, Tokyo, Japan; 3 Department of Clinical Research and Informatics, International Clinical Research Center, Research Institute, National Center for Global Health and Medicine, Tokyo, Japan; 4 Department of Infectious Disease, National Center for Global Health and Medicine, Tokyo, Japan; 5 Division of AIDS Clinical Center (ACC), National Center for Global Health and Medicine, Tokyo, Japan; Institut National de la Santé et de la Recherche Médicale, France

## Abstract

**Background:**

Candidia esophagitis (CE) is an AIDS-defining condition, usually occurring in individuals with low CD4 counts of <200 cells/µL. Endoscopy is a valuable definitive diagnostic method for CE but may not be indicated for asymptomatic patients or for those with high CD4 counts or without oral candidiasis. This study assessed such patients to clarify the factors associated with CE and its severity on endoscopy in the highly active antiretroviral therapy (HAART) era.

**Methodology/ Principal Findings:**

A total of 733 HIV-infected patients who underwent upper gastrointestinal (GI) endoscopy were analyzed. Sexual behavior, CD4^+^ count, HIV-RNA viral load (VL), history of HAART, GI symptoms, GI diseases, and oral candidiasis were assessed. Endoscopic severity of CE was classified as mild (Kodsi's grade I/II) or severe (grade III/IV). Of the 733 subjects, 62 (8.46%) were diagnosed with CE (mild, n = 33; severe, n = 29). Of them, 56.5% (35/62) had no GI symptoms, 30.6% (19/62) had CD4 + ≥200 cells/μL, and 55.3% (21/38) had no oral candidiasis. Univariate analysis found lower CD4+ counts, higher HIV VL, and no history of HAART to be significantly associated with CE. With lower CD4^+^ counts and higher HIV VL, CE occurrence increased significantly (*P*<0.01 for trend in odds). Multivariate analysis showed low CD4+ counts and high HIV VL to be independently associated with CE. Of the severe CE patients, 55.2% (16/29) had no GI symptoms and 44.4% (8/18) had no oral candidiasis. Median CD4^+^ counts in severe cases were significantly lower than in mild cases (27 vs. 80; *P* = 0.04).

**Conclusions:**

Low CD4+ counts and high HIV VL were found to be factors associated with CE, and advanced immunosuppression was associated with the development of severity. Endoscopy is useful as it can detect CE, even severe CE, in patients without GI symptoms, those with high CD4 counts, and those without oral candidiasis.

## Introduction

Candida esophagitis (CE) is the most common infectious disease of the esophagus [1]–[3] and the most common gastrointestinal (GI) opportunistic disorder among individuals infected with human immunodeficiency virus (HIV) [4], [5]. The prevalence of CE among HIV patients with esophageal symptoms who have undergone endoscopy was approximately 42% prior to the introduction of highly active antiretroviral therapy (HAART) [6]. Although the rate of AIDS-related opportunistic infections has decreased dramatically since, CE remains one of the most common AIDS defining illnesses [4]–[6].

Only a limited number of studies have investigated the associated factors of CE, and no study has yet been conducted with a large population of patients. In the pre-HAART era, a low CD4+ cell count (<200 cells/μL) was identified as one such associated factor [1], [3], [5], [7], [8]. In the HAART era, esophageal symptoms such as odynophagia and dysphagia are associated factors, while a CD4^+^ cell count >200, an HIV viral load <400, and the presence of gastric ulcers are considered protective factors for CE [6]. The prevailing view is that a presumptive diagnosis of CE can usually be made following the recent onset of typical symptoms [7] or the presence of oral candidiasis, and empiric antifungal therapy can then be started as a diagnostic trial [7], [9]–[11].

Endoscopy is a highly valuable definitive method for CE as it enables tissue biopsy and pathological and cultural examinations of specimens in addition to the evaluation of gross appearance [7], [8], [10], [12]. However, under these conditions, endoscopy may not be indicated for asymptomatic patients, or for those with high CD4 cell counts or without oral candidiasis. The gross appearance and severity of CE on endoscopy can vary from small white plaques or confluent, linear, and nodular elevated plaques to thick white plaque cover on esophageal mucosa which may cause circumferential narrowing of the esophageal lumen [7], [8], [10], [12]. However, the clinical factors associated with CE severity remain to be identified. In Japan, screening endoscopy is frequently performed for the early detection of malignant or premalignant lesions, even for asymptomatic patients. In this study, we performed endoscopy for a large number of HIV-infected patients including those with no GI symptoms, high CD4 counts, or no oral candidiasis.

## Materials and Methods

### Objectives

We conducted a cross-sectional study to identify clinical factors associated with the diagnosis of CE. We also assessed the association between the severity of CE and clinical factors.

### Participants

Upper gastrointestinal endoscopy was performed in a cohort of 752 HIV-infected patients between 2003 and 2009 at the National Center for Global Health and Medicine (NCGM), a 900-bed hospital located in the Tokyo metropolitan area and the largest referral center for HIV/AIDS in Japan.

Indications for endoscopy were: 1) gastrointestinal symptoms in symptomatic patients; and 2) fecal occult blood test results, abnormal findings on CT or annual health checkups, or laboratory abnormal findings of tumor markers or infections suspicious of colorectal cancer in asymptomatic patients. In Japan, screening endoscopy is frequently performed for the early detection of malignant or premalignant lesions, even as part of the examination for patients who are asymptomatic.

We excluded patients who had received endoscopy for follow-up evaluation shortly after treatment for GI diseases. Written informed consent was obtained from all patients prior to endoscopy and biopsy. Study protocols were approved by the NCGM ethics committee.

### Diagnosis of Candida Esophagitis

CE was suspected on endoscopic findings on the basis that candidal white plaques in the esophagus cannot be washed away [13]. Biopsy was performed when endoscopy revealed white plaques in the esophagus. For pathological diagnosis, hematoxylin and eosin (HE) and periodic acid-Schiff (PAS) staining were used to identify candida. In the case of negative CE biopsy results, visible CE lesions on endoscopy were confirmed to be resolved following antifungal therapy.

### Endoscopic Assessment

**Figure 1:**
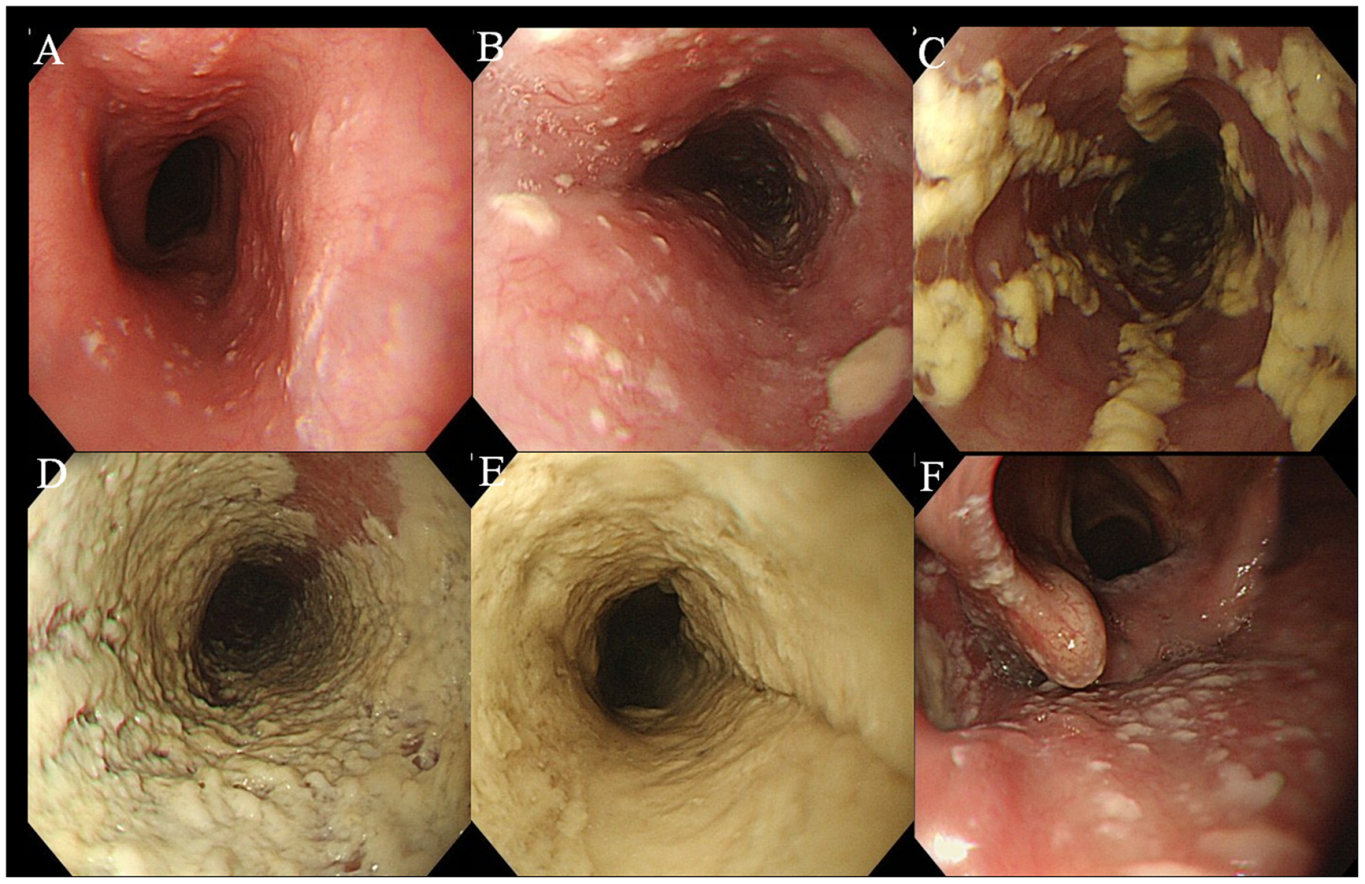
Endoscopic severity of Kodsi's grading. A: Grade I, a few raised white plaques up to 2 mm in size without edema or ulceration. B: Grade II, multiple raised white plaques greater than 2 mm in size without ulceration. C: Grade III, confluent, linear, and nodular elevated plaques. D: Grade IV, finding of grade III with increased friability of the mucous membranes and occasional narrowing of the lumen. E: “White carpet” appearance, thick white plaque cover on esophageal mucosa circumferential narrowing the lumen. F: Oral Candidiasis, in which endoscopy can detect laryngopharyngeal candidiasis.

Endoscopy was performed by gastroenterologists at NCGM with a high resolution gastroscope (GIF-H260 or Q260, Olympus Corp., Japan). Gross appearance on endoscopy was graded according to Kodsi's classification as grade I–IV, with grade I/II defined as mild and grade III/IV defined as severe (Figure 1A–D) [14]. In addition, an endoscopic finding of candidal white plaques covering the entire circumference of the esophageal lumen was referred to as “white carpet” (Figure 1E). The presence/absence of oral candidiasis was determined by oral endoscopic imaging (Figure 1F) and medical record descriptions. Endoscopic Kodsi classification grades were assessed by two physicians (S.N. and N.A.), and interobserver agreement for the endoscopic findings was evaluated. Two physicians made their judgments from endoscopic images only and were blinded to the subjects' clinical details (HIV results, HIV-RNA, CD4 counts, symptoms, and histological results). Only endoscopic findings obtained by one observer (N.A.) were used for evaluation.

### Clinical Factors

GI symptoms were collected from medical records written by an attending doctor who interviewed each patient before endoscopy. Those without GI symptoms or descriptions of symptoms in their medical charts and those with a reason for receiving “screening” endoscopy were treated as asymptomatic cases.

GI symptoms comprised epigastralgia, nausea, heart burn, dysphagia, throat pain, appetite loss, hematemesis, and tarry stools. CD4^+^ cell count values obtained within 2 weeks before or after endoscopy were collected from patient medical records and categorized into the following three groups: ≥200 cells/μL; 100–199 cells/μL; <100 cells/μL. HIV-RNA viral load (VL) as determined by real-time quantitative polymerase chain reaction (PCR) was also reviewed within 1 month of endoscopy. The minimum detection level was 40 copies/mL of plasma, and a positive result was defined as ≥40 copies/mL. We categorized HIV-RNA VL into four groups: VL ≤40 copies/ml (normal range); 40<VL≤10,000 copies/ml; 10,000<VL≤100,000 copies/ml; and 100,000< VL copies/ml.

Routes of infection were determined using face-to-face questionnaires conducted by the medical staff. HIV infection routes were classified into the following five categories: men who have sex with men (MSM); heterosexual; drug user; use of untreated blood products; and unknown. Sexual behavior was divided into two categories: MSM and heterosexual. Patients who were neither homosexual nor bisexual were regarded as heterosexual.

The presence of HIV-related GI diseases such as cytomegalovirus (CMV), herpes simplex virus (HSV), Kaposi's Sarcoma (KS), *Mycobacterium avium* complex (MAC), malignant lymphoma (ML), and idiopathic ulcer, as well as other GI diseases such as esophageal varix, gastric adenoma, gastric adenocarcinoma, and reflux esophagitis were verified by examination of medical records and the endoscopic database. Information regarding history of HAART was collected from pre-endoscopy medical records.

### Statistical analysis

The odds ratio (OR) and 95% confidence interval (CI) were used to determine factors associated with CE, and the relationships between CE and CD4^+^ cell count and HIV-RNA viral load were evaluated using the Chi-square test for linear trends. A multiple logistic regression model was used in multivariate analysis with factors showing values of *P*<0.2 on univariate analysis. A final model was then developed by backward selection of factors showing values of *P*<0.1. To identify the best performing combination of clinical factors, the area under the receiver operating characteristic curve (ROC-AUC) was calculated.

The interobserver agreement of endoscopic severity (mild or severe) was measured with kappa statistics. Kappa values >0.80 were denoted excellent, 0.60–0.80 good, 0.40–0.60 moderate, 0.20–0.40 fair, and <0.20 poor.

Association between the severity of CE and clinical factors were evaluated. The Mann-Whitney U test was used for age, CD4^+^ cell count, and HIV-RNA viral load. Fisher's exact test was used for sex, sexual behavior, history of HAART, and the presence of GI symptoms and oral candidiasis. Values of *P*<0.05 were considered significant, and all statistical analysis was performed using Stata version 10 software (StataCorp LP, College Station, TX).

## Results

### Participants

Of the 752 potential study subjects we recruited who underwent endoscopy, 19 patients who underwent endoscopy for follow-up evaluation shortly after treatment for GI diseases were excluded. The remaining 733 patients were selected for data analysis.

### Baseline Characteristics

Patient characteristics are summarized in Table 1. The median age was 44 years (interquartile range [IQR], 36–56 years), and patients were predominantly male (92.8%). Routes of HIV infection included MSM (62.9%), heterosexual (18.1%), hemophilia (16.8%), drug use (0.3%), and unknown (1.9%). The median CD4^+^ cell count was 234 cells/μL (IQR, 97–399 cells/μL), and the median HIV-RNA viral load was <40 copies/mL (IQR, <40–23,000 copies/mL). HAART had been administered to 545 patients (74.35%). The median CD4^+^ cell count was significantly higher in patients with HAART than in those without (265 vs 121 cells/μL, *P*<0.01). The median HIV-RNA viral load was significantly lower in patients with HAART than in those without (<40 vs 45,000 copies/mL, *P*<0.01).

**Table 1:** Patient characteristics (n = 733).

Characteristic	Number (%)
Age, median (IQR)	44 (36, 56)
Sex (male), n (%)	680 (92.8%)
HIV infection route, n (%)	
MSM	461 (62.9%)
Heterosexual	133 (18.1%)
Hemophilia	123 (16.8%)
Drug use	2 (0.3%)
Unknown	14 (1.9%)
CD4^+^ cell count, median (IQR) (cells/μL)	234 (97, 399)
≥200	407 (55.5%)
100–199	138 (18.8%)
<100	188 (26.7%)
HIV-RNA VL, median (IQR) (copies/mL)	<40 (<40, 23000)
≤40 (normal range)	386 (52.7%)
40<VL≤10,000	136 (18.6%)
10,000<VL≤100,000	99 (13.5%)
100,000< VL	112 (15.3%)
History of HAART, n (%)	545 (74.4%)
With GI symptoms, n (%)	263 (35.9%)

Abbreviations: MSM, men who have sex with men; IQR, interquartile range; VL, viral load; HAART, highly active anti-retroviral therapy; GI, gastrointestinal.

GI symptoms were noted in 263 patients (35.9%) and included epigastalgia (n = 84), nausea (n = 36), tarry stool (n = 29), hematemesis (n = 25), heart burn (n = 22), dysphagia (n = 12), throat pain (n = 8), and appetite loss (n = 5). The endoscopic diagnosis of upper GI diseases is shown in Table 2. Of the 733 patients, 62 (8.46% [95% CI, 6.54–10.71]) were diagnosed with CE. The presence or absence of oral candidiasis was determined in 38 patents from endoscopic images, of which 17 were found to be positive.

**Table 2:** Upper gastrointestinal diseases (n = 733).

HIV-related disease		Number (%)
Candida esophagitis		62 (8.5%)
Cytomegalovirus GI disease	32 (4.4%)	
Herpes esophagitis	1 (0.1%)	
MAC infection	3 (0.4%)	
Kaposi's Sarcoma		44 (6.0%)
Malignant lymphoma		19 (2.5%)
HIV-related idiopathic Ulcer		2 (0.3%)
Others		
Esophageal Varix		89 (12.1%)
Reflux esophagitis		40 (5.5%)
Gastric adenoma		3 (0.4%)
Gastric adenocarcinoma		3 (0.4%)

Abbreviations: GI, gastrointestinal; MAC, mycobacterium avium complex.

### Clinical Factors Associated with CE

Of all CE patients, 56.5% (35/62) had no GI symptoms, 30.6% (19/62) had CD4 +≥200 cells/μL, and 55.3% (21/38) had no oral candidiasis (Table 3). Univariate analysis revealed that a low CD4^+^ cell count, higher HIV-RNA viral load, and no history of HAART were significantly associated with CE (Table 3).

**Table 3:** Clinical factors for candida esophagitis on uni- and multivariable analysis (n = 733).

Clinical factors	CE (+) (n = 62)	CE (−) (n = 671)	Odds ratio (95% CI)	*P*
**Univariate analysis**				
Age (years)				
<40	21	236	1 (referent)	
≥40	41	435	1.06 (0.60–1.93)	0.84
Sex				
Female	7	46	1 (referent)	
Male	55	625	0.58 (0.24–1.59)	0.19
Sexual behavior				
Heterosexual	23	249	1 (referent)	
MSM	39	422	1.00 (0.57–1.80)	1.00
CD4^+^ cell count (cells/μL)				
>200	19	388	1 (referent)	
100-199	4	134	0.61 (0.20–1.82)	
<100	39	149	5.35 (2.99–9.55)	<0.01
HIV-RNA VL (copies/mL)				
≤40 (normal range)	10	376	1 (referent)	
40<VL≤10,000	13	123	3.97 (1.70–9.29)	
10,000<VL≤100,000	15	84	6.71 (2.91–15.5)	
100,000< VL	24	88	10.3 (4.73–22.2)	<0.01
History of HAART				
Without	30	158	1 (referent)	
With	32	513	0.33 (0.19–0.58)	<0.01
GI symptoms				
Without	35	435	1 (referent)	
With	27	236	1.42 (0.81–2.48)	0.18
**Multivariate analysis**				
CD4^+^ cell count (cells/μL)				
>200	19	388	1 (referent)	
100-199	4	134	0.43 (0.14–1.30)	
<100	39	149	2.82 (1.46–5.46)	<0.01
HIV-RNA VL (copies/mL)				
≤40 (normal range)	10	376	1 (referent)	
40<VL≤10,000	13	123	3.67 (1.54–8.74)	
10,000<VL≤100,000	15	84	5.34 (2.24–12.7)	
100,000< VL	24	88	5.67 (2.39–13.4)	<0.01

Abbreviations: CE, Candida esophagitis; MSM, men who have sex with men; VL: viral load; HAART, highly active antiretroviral therapy.

With a lower CD4^+^ count (≥200; 100–199; and <100 cells/μL), the occurrence of CE increased significantly (*P*<0.01 for trend in odds, Table 3). Similarly, with a higher HIV-RNA VL (VL≤40; 40<VL≤10,000; 10,000<VL≤100,000; and 100,000<VL copies/ml), the occurrence of CE increased significantly (*P*<0.01 for trend in odds, Table 3).

Multivariate analysis revealed that lower CD4^+^ cell counts and a higher HIV-RNA VL were independent associated factors for CE (Table 3). Performance of the combination of these two factors was calculated as 0.78 (95% CI, 0.72–0.83) in ROC-AUC analysis.

### Endoscopic Severity and Clinical Factors

Endoscopy placed 33 patients in the mild group (grade I/II: 7/26) and 29 in the severe group (grade III/IV: 24/5). A “white carpet” was observed in three grade-IV patients. The interobserver agreement for endoscopic severity was shown to be 0.77 (rated as “good”) by Kappa statistics.

Table 4 summarizes the distribution of clinical factors of CE patients by severity. Among the severe CE patients, 55.2% (16/29) had no GI symptoms and 44.4% (8/18) had no oral candidiasis. No significant associations were found between groups with respect to age, sex, percentage of MSM, HIV-RNA viral load, history of HAART, GI symptoms, and oral candidiasis. The median CD4^+^ cell count in severe cases was significantly lower than in mild cases (27 vs. 80; *P* = 0.0374).

**Table 4:** Endoscopic severity and clinical factors for candida esophagitis (n = 62).

Factor	Mild (n = 33)	Severe (n = 29)	*P*
Age^a^	46 (33, 67)	41 (28, 59)	0.18^c^
Sex (male)	30 (90.9%)	25 (86.2%)	0.56
Sexual behavior (MSM)	21 (63.6%)	18 (62.1%)	1.00^d^
CD4+ cell count (cells/μL)^a^	80 (12, 425)	27 (9, 226)	0.04^d^
HIV-RNA viral load (copies/mL) a	3.8×10^4^ (<40, 6.5×10^5^)	6.3×10^4^ (<40, 2.2×10^5^)	0.90^c^
History of HAART	16 (48.5%)	16 (55.2%)	0.60^d^
With GI symptoms	14 (42.4%)	13 (44.8%)	0.85^d^
With oral candidiasis^b^	7/20 (35%)	10/18 (55.6%)	0.38^d^

a Median (interquartile range).

b The existence of oral candidiasis was checked in 38 patients by endoscopy.

c *P* for Mann-Whitney U test; ^d^ *P* for Fisher's exact probability test.

Abbreviations: MSM, men who have sex with men; HAART, highly active antiretroviral therapy.

## Discussion

CE is the most common esophageal disorder observed in HIV patients [15], with a prevalence of 43–53% in the pre-HAART era that has decreased to 17–24% since the introduction of HAART [6], [8], [16]. In the present study, CE was found in 8.46% of HIV patients who underwent upper GI endoscopy. This is lower than reported previously, probably because the present study included many asymptomatic patients.

The presence of CE is an indication for systemic treatment with antifungals [11], and endoscopy is essential for diagnosing CE. Endoscopy is usually performed as a diagnosis for patients with GI symptoms, but a previous study reported HIV patients with CE who were totally asymptomatic [5]. Surprisingly, the present patient population included 35 (56.1%) CE patients with no GI symptoms. These observations suggest the need for identifying clinical factors other than GI symptoms predictive of CE.

In the present study, univariate analysis identified the absence of prior HAART, low CD4^+^ cell count, and higher HIV-RNA viral load as factors associated with CE. Multivariate analysis identified a low CD4^+^ cell count and a higher HIV-RNA VL as independent associated factors. A low CD4^+^ cell count and high HIV-RNA viral load as associated factors are consistent with previous reports. López-Dupla and colleagues [5] compared several factors between patients with oral candidiasis and those with CE in the pre-HAART era and found that the CD4:CD8 ratio was significantly lower in CE patients and that lymphocyte and CD4^+^ cell counts were lower, albeit non-significantly, in CE patients. Their study included a large number of patients (n = 114) but, as a case series, did not compare the data with those obtained from control patients [5]. Nkuize and colleagues [6] studied 706 patients infected with HIV (239 from the pre HAART era, 238 from the early HAART era, and 229 from the recent HAART era). Of the 238 patients from the early HAART era, they identified odynophagia/dysphagia as an associated factor and an HIV-RNA viral load <400 copies/mL, a CD4^+^ cell count >200 cells/μl, and gastric ulcers as protective factors. However, their study did not consider the history of HAART or the presence of asymptomatic patients. Manfredi and colleagues [17] speculate that high HIV-RNA VL is an important factor associated with the recurrence of CE, regardless of immune status.

CE is classified into mild and severe cases according to endoscopic findings, and severe cases are associated with complications such as hemorrhage, stenosis, and esophagotracheal fistula [18], [19]. It is therefore important to identify clinical factors associated with severe CE cases, and we identified a low CD4^+^ cell count as one such factor. Some patients with severe CE had characteristic endoscopic findings such as “white carpet” (Figure 1E), which has not been reported previously, and suggests the involvement of compromised immune function in its formation. The presence of GI symptoms was not associated with severe CE. More surprisingly, 55.2% of patients with severe CE were asymptomatic, indicating that the determination of endoscopic indications on the basis of symptoms alone may lead to a missed diagnosis of severe CE.

Werneck-Silva and colleagues [15] demonstrated that Kodsi's grade-I (mild) cases are associated with significantly higher CD4^+^ cell counts than other grades while there is no significant difference in CD4^+^ cell count between grade II–IV cases. Wilcox and colleagues [10] also reported that many CE patients endoscopically graded as severe had a CD4^+^ cell count <200, although their data are inadequate as their study included only a small number of HIV patients with CE. We also evaluated oral candidiasis, in addition to clinical factors, but found no association with the severity of CE.

There are several limitations in the present study. Although oral candidiasis is considered an important associated factor for the diagnosis of CE [7], [9], [13], this finding was not observed in all patients in the present study, and some patients were missing relevant oral candidiasis data. Second, we did not evaluate the clinical factors of diabetes and malignancy, or the use of oral antibiotics and antacids as associated factors for CE in non-HIV patients [15], [20]–[22]. Third, selection bias was present, for although subjects were patients who underwent endoscopy, those who had severe clinical conditions such as unstable circulatory or respiratory dynamics or bleeding tendency may have been more likely to be excluded. Therefore, the present results are applicable to patients with relatively good general condition.

## Conclusions

Clinical factors should be considered when determining the endoscopic indication to diagnose CE. Low CD4^+^ cell counts and higher HIV-RNA VL were identified as clinical factors associated with CE, while higher endoscopic severity was shown to be associated with lower CD4^+^ cell counts. Endoscopy is useful as it can detect CE, even severe CE, in patients without GI symptoms, those with high CD4 counts, and those without oral candidiasis.
